# Classical, Quantum and Event-by-Event Simulation of a Stern–Gerlach Experiment with Neutrons

**DOI:** 10.3390/e24081143

**Published:** 2022-08-17

**Authors:** Hans De Raedt, Fengping Jin, Kristel Michielsen

**Affiliations:** 1 Institute for Advanced Simulation, Jülich Supercomputing Centre, Forschungszentrum Jülich, D-52425 Jülich, Germany; 2 Zernike Institute for Advanced Materials, University of Groningen, 9747 AG Groningen, The Netherlands; 3 Department of Physics, RWTH Aachen University, D-52074 Aachen, Germany

**Keywords:** Stern–Gerlach experiment, classical mechanics, quantum mechanics, event-by-event simulation

## Abstract

We present a comprehensive simulation study of the Newtonian and quantum model of a Stern–Gerlach experiment with cold neutrons. By solving Newton’s equation of motion and the time-dependent Pauli equation for a wide range of uniform magnetic field strengths, we scrutinize the role of the latter for drawing the conclusion that the magnetic moment of the neutron is quantized. We then demonstrate that a marginal modification of the Newtonian model suffices to construct, without invoking any concept of quantum theory, an event-based subquantum model that eliminates the shortcomings of the classical model and yields results that are in qualitative agreement with experiment and quantum theory. In this event-by-event model, the intrinsic angular momentum can take any value on the sphere, yet, for a sufficiently strong uniform magnetic field, the particle beam splits in two, exactly as in experiment and in concert with quantum theory.

## 1. Introduction

In 1922, O. Stern and W. Gerlach demonstrated experimentally that silver atoms passing through an inhomogeneous magnetic field experience deflections in spatially different, distinguishable directions. This observation was very important for the early development of quantum theory for it provided direct experimental evidence that not only the spectra of atoms but also the magnetic moment of the particles might be quantized [1,2,3]. The Stern–Gerlach (SG) experiment is often used in textbooks [4,5,6,7] to introduce the concepts of spin and quantization of angular momentum and plays a prominent role in discussions on determining properties of atomic size objects by means of macroscopic measuring devices [8,9]. The SG experiment, and its conceptually equivalent experiment with single photons passing through a birefringent crystal, are also used in textbooks to illustrate postulates of quantum theory [4,5,6,7].

**Figure 1:**
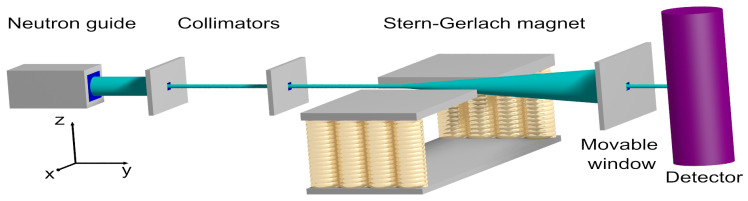
Diagram (not to scale) of a Stern–Gerlach experiment with cold neutrons, performed by Hamelin et al. [10]. After passing through the collimators, most neutrons travel along the *y*-direction. The cone indicates the directions in which the neutron may, but not necessarily have to, leave the magnetic field region.

In short, an SG experiment involves a source of electrically neutral, magnetic particles, collimators, a magnet generating an inhomogeneous field, and a particle detector; see Figure 1 for a sketch of an SG with cold neutrons. Due to the interaction between the magnetic moment of the particle and the inhomogeneous magnetic field, a particle passing through the latter experiences a force that changes the trajectory of the particle. Note that this reasoning is entirely Newtonian, no concept of quantum theory is entering yet.

Assuming (i) uniformly random orientations of the magnetic moments leaving the source and (ii) a sufficiently large uniform magnetic field, the standard classical picture of the magnetic moment as a spinning top leads to the conclusion that there should be no splitting of the beam [5]; however, under certain conditions [11], to be scrutinized in the present paper, the SG magnet splits the particle beam in two, spatially well-separated directions, in agreement with the outcome of the SG experiment. As the amount of deflection is proportional to the magnetic moment, an SG-like apparatus can be used to measure the magnetic moment of nano-size particles [12,13].

As originally conceived, the SG experiment employs electrically neutral particles. Obviously, this begs the question if it would be feasible to perform a similar experiment to observe the spin of say, electrons [14,15,16,17] or ions [18]. Addressing this interesting question is beyond the scope of the present paper, which focuses on the case of electrically neutral particles only.

The deflection in spatially well-separated directions along the direction of the uniform magnetic field is commonly regarded as an experimental proof that the magnetic moment of the particles is quantized [1,2,4,5]. Labeling the distinct beams by a two-valued variable s=±1/2 and representing the beams by the corresponding state vectors forms the basis for the well-known quantum-theoretical description of the idealized SG experiment [4,5,6,7,19,20].

The first aim of the present paper is primarily pedagogical in that we present, to the best of our knowledge, the first comprehensive treatment of both the Newtonian and quantum model of a real SG experiment. In order to touch base with a real SG experiment, we have taken model parameters from an SG experiment performed with cold neutrons [10]. In this respect, there is little overlap with earlier numerical studies of the quantum model of an SG experiment [21,22].

The second aim is to demonstrate that a minor modification to the classical, Newtonian equations of motion in the spirit of the event-by-event simulation approach yields results that (i) can be very different from those of the classical and (ii) are in full qualitative agreement with SG experiments and with the quantum-theoretical description thereof. The idea behind this modification is the following. As long as the particle does not experience a magnetic field, the internal frame of reference used to define the direction of the magnetic moment is detached from the laboratory frame of reference. This hold true in quantum theory as well: in the absence of an electromagnetic field there is no relation between the xyz-coordinates of the particle and xyz-components of the spin operator [7]. In the event-based approach, a particle moving from a field-free region into a region where the electromagnetic field is present is viewed as an event which establishes the relation between the xyz-coordinates of the particle and xyz-components of the magnetic moment. This event-triggered process of alignment may be thought of as a highly simplified model for the classical electrodynamic transient processes that occur when a magnetic moment moves through a region in which the magnetic field changes [23].

The paper is structured as follows. Section 2 describes the SG experiment with neutrons [10] that we take as reference for our simulation work. In Section 3 and Section 4, we present and discuss the results obtained by solving Newton’s equation of motion and the time-dependent Pauli equation (TDPE), respectively. Adopting the parameters for the cold neutrons SG experiment in combination with the macroscopic size of the experimental setup requires the use of high-precision solvers and substantial computer resources. In Section 4, we also discuss the transition from a description in terms of position and spin to a model that involves spin-1/2 operators only. Section 5 introduces the modification to Newton’s equation of motion that turns the classical model into a event-by-event, subquantum model for the SG experiment, meaning that data generated by the latter exhibit the same features as the data obtained by SG experiments and their quantum-theoretical description. Section 6 summarizes our findings.

## 2. Neutron Experiment

Figure 1 shows a schematic of the SG experiment with neutrons, as performed by Hamelin et al. [10]. Cold neutrons leaving the neutron guide impinge on a collimator positioned 0.2m from the exit plane of the neutron guide. The selected neutrons impinge on a second collimator, placed 1m from the first one. The strongly collimated beam of neutrons then passed through the SG magnet which is 0.8m long. The distance between the second collimator and the exit plane of the SG magnet is 0.9m. The direction of the neutrons leaving the SG magnet is selected by means of a meaning window. The distance between the exit plane of the SG magnet and the ${}^{3}\mathrm{He}$ detector is 2m.

**Figure 2:**
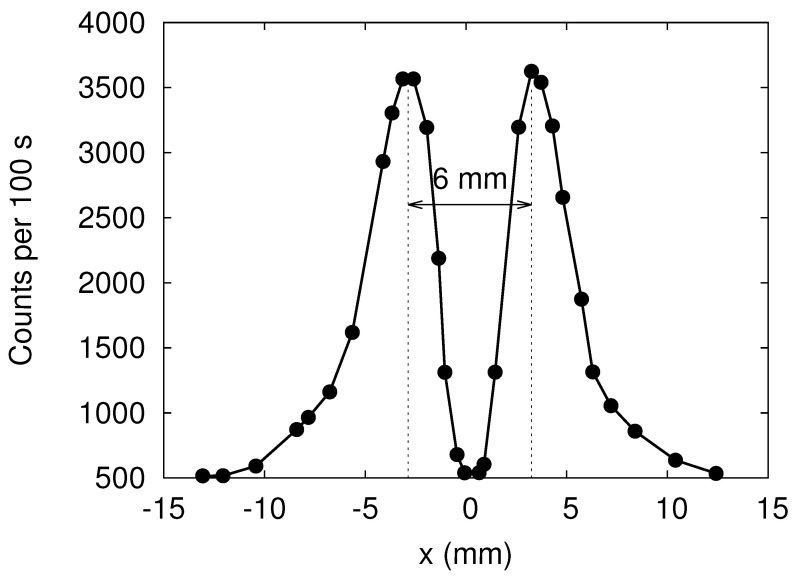
Neutron counts per 100 s as recorded in the SG experiment by Hamelin et al. [10]. The data were extracted from Figure 6 of Ref. [10] by hand.

In Figure 2 we present some of the results reported in Ref. [10]. Clearly, the SG magnet causes the neutron beam to split in two well-defined beams, with their maxima of intensities separated by about 6mm. Note that the window (see Figure 1) in front of the detector moves in the *x*-direction only.

Looking at the experimental data presented in Figure 2, it is obvious that in order to represent the spin state of a neutron by a two-valued variable, it is necessary to classify the data points as belonging to one of two groups. As the two maxima of the counts are well separated, simply drawing a vertical line at x=0 suffices to classify the data points. Once this classification is made, we can dispose of the spatial degree of freedom and describe the process of spin-based filtering in terms of spin-1/2 matrices, a model that is often used in textbooks [4,5,6,7].

## 3. Newtonian Mechanics

The Hamiltonian describing the dynamics of a neutral, particle of mass *m* and magnetic moment M subject to a time-independent magnetic field B=B(x) reads

(1) $$\begin{array}{ccc} H & = & \frac{m}{2}v^{2}-M\cdot B\left(x\right)\;=\frac{m}{2}v^{2}-\gamma \;L\cdot B\left(x\right)\;, \end{array}$$

where L is the angular momentum relative to the center of mass x of the particle, and γ is the gyromagnetic ratio. Starting from Equation (1), the standard procedure to derive the equations of motion yields,

(2) $$\begin{array}{ccc} m\frac{dv}{dt} & = & \gamma \;\nabla \;(B(x)\cdot L)\;, \end{array}$$

(3) $$\begin{array}{ccc} \frac{dL}{dt} & = & \gamma \;L\times B(x)\;. \end{array}$$

The angular momentum L has the same dimension as *ℏ*, namely $\left(\mathrm{kg}\;m^{2}\;s^{-1}\right)$. In order to facilitate the comparison with the quantum-theoretical description, it is expedient to define L=ℏS where $S=S{\left(\cos\phi \sin\theta ,\sin\phi \sin\theta ,\cos\theta \right)}^{T}$ is a dimensionless vector. In terms of this vector, the classical equations of motion read

(4) $$\begin{array}{ccc} \frac{m}{\hbar }\frac{dv}{dt} & = & \gamma \;\nabla \;(B(x)\cdot S)\;, \end{array}$$

(5) $$\begin{array}{ccc} \frac{dS}{dt} & = & \gamma \;S\times B(x)\;. \end{array}$$

Note that the presence of *ℏ* is the result of rewriting the classical equations of motion in terms of a dimensionless angular momentum S and does not, in any way, imply that Equations (4) and () describes quantum phenomena. The length *S* of the vector S does not affect the solution of Equation (5) and needs to be fixed by comparison with the results of the quantum-theoretical description; this is described in a later section of the paper.

### 3.1. Model for the Magnetic Field

Essential to an SG experiment is that the magnetic particles interact with an *inhomogeneous* magnetic field. Maxwell’s equation requires that ∇·B(x)=0. From the Maxwell equation

(6) $$\begin{array}{c} \frac{\partial E(x,t)}{\partial t}=\frac{1}{\epsilon \mu }\nabla \times B\left(x\right)-\frac{1}{\epsilon }J\left(x,t\right)\;, \end{array}$$

where J(x,t), ϵ and μ represent the external current, the electrical permittivity, and magnetic permeability, respectively. It follows that if ∇×B(x)≠0, the magnetic field would induce a nonzero, time-dependent electric field E(x,t). The strength of this electric field would increase linearly with time. Although this electric field would not affect the motion of the electrically neutral particles, in our study, we only consider the case ∇×B(x)=0.

A simple choice, complying with the conditions ∇·B(x)=0 and ∇×B(x)=0 just mentioned, is [11,21,22,24,25,26]

(7) $$\begin{array}{ccc} B(x) & = & \left\{\begin{array}{cc} \left(B_{0}+zB_{1}\right)e_{z}-xB_{1}e_{x} & \;,\;y\in [y_{0},y_{1}]\\ 0 & \;,\;y\notin [y_{0},y_{1}] \end{array}\right.\;, \end{array}$$

that is, B(x)=0 except when $y_{0}\leq y\leq y_{1}$ where the strength of the field gradient in both the *x* and *z* direction is $B_{1}>0$ (we adopt the convention that $B_{0},B_{1}\geq 0$). The term in Equation (7) proportional to $B_{0}$, the uniform magnetic field in the *z*-direction, describes the contribution of the dipole field. The two terms in Equation (7) proportional to $B_{1}$ are characteristic for the quadrupole contribution to the magnetic field. The values of $B_{0}$ and $B_{1}$ depend on the design of the magnet. In this paper, we regard $B_{0}$ and $B_{1}$ as model parameters.

From Equation (7) it follows that for $y\in [y_{0},y_{1}]$, the force F(x) on the particle is given by

(8) $$\begin{array}{ccc} F(x) & = & \gamma \;\nabla \;\left(B\left(x\right)\cdot S\right)=\gamma \;B_{1}\left(S^{z}e_{z}-S^{x}e_{x}\right)\;, \end{array}$$

independent of *x* or *z*. For $y\notin [y_{0},y_{1}]$, the force F(x) on the particle is zero. As a function of *y*, the simple model Equation (7) shows discontinuities at $y=y_{0},y_{1}$. Instead of smoothing out these discontinuities, we integrate the equations of motion in the interval $y_{0}\leq y\leq y_{1}$ and assume that the velocity distribution at $y=y_{1}$ is representative (up to trivial, free-particle scale factors) for the velocity and position distributions at $y\gg y_{1}$.

From Equation (8), it follows immediately that the velocity in the *y*-direction is conserved. In this paper, we assume that all particles move with velocity $v_{y}$ along the *y*-direction. The time it takes for the particles to traverse the magnetic field region is given by $t^{*}=\left(y_{1}-y_{0}\right)/v_{y}$.

Once a particle’s *y*-coordinate exceeds $y_{1}$, its velocity $v=(v_{x},v_{y},v_{z})$ is used to increment the histogram count at the transverse velocity coordinate $\left(v_{x},v_{z}\right)$ and the simulation of that particle is terminated. The distribution of transverse velocities $\left(v_{x},v_{z}\right)$ does not change if the particles leave the region where the magnetic field is present and is therefore well-suited to analyze the data. The distribution of transverse positions (x,z) at any plane located to the right of the SG (see Figure 1) is straightforwardly obtained from the distribution of transverse velocities by using the fact that in the field-free region, the particles propagate freely.

In this paper, we mainly present results for the distribution of the transverse velocities $\left(v_{x},v_{z}\right)$, obtained by classical, quantum-theoretical, and event-by-event simulation. This distribution contains all information about the outcome of the simulated SG experiment and facilitates the presentation of the simulation data in a compact, unified, and convenient manner.

### 3.2. Analytically Solvable Cases

It is of interest to consider a special case that is easy to solve analytically. We take as initial positions and velocities of the *N* particles $x=(0,y_{0},0)$ and $v=(0,v_{y},0)$, respectively, and we only consider the case in which all particles have their initial magnetic moment along the *z*-axis, i.e., S=S(0,0,±1). Note that S×B(x=(0,y,z))=0 for any (y,z), see Equation (7), implying that for x=(0,y,z), the torque on the spin is zero; therefore, the direction of the spin does not change and the particles only feel a constant force in the *z*-direction, see Equation (8). The trajectory is that of a particle in a constant force field, that is $v_{z}\left(t\right)=\pm \hbar \gamma B_{1}St/m$ and $z\left(t\right)=\pm \hbar \gamma B_{1}St^{2}/2m$ for $0\leq t\leq t^{*}$.

Looking ahead, this simple scenario mimics the quantum-theoretical textbook case (see Appendix D.2) and allows us to fix the magnitude of the classical magnetic moment S. Indeed, the classical and quantum-theoretical expressions for the change in the velocity due to the magnetic field gradients match if S=1/2.

From the analysis of the analytically solvable, classical mechanical case, it follows that the time of flight, the changes of transverse velocity and displacement are given by

(9) $$t^{*}=\frac{y_{1}-y_{0}}{v_{y}}\;,\;v^{*}=\left|\frac{\hbar \gamma B_{1}}{2m}\right|t^{*}\;,\;z^{*}=\frac{v^{*}t^{*}}{2}\;,$$

respectively. The three parameters Equation (9) characterize the state of the particles at the point $y=y_{1}$, that is when they leave the region where the magnetic field is present. Again, looking ahead, the quantum-theoretical textbook case also yields Equation (9). We use Equation (9) to set the scale of time, velocity, and position for both the classical and quantum-theoretical model.

The second solvable case is the one that is often referred to when comparing the classical and quantum-theoretical picture of the magnetic moment.

If the uniform magnetic field is present ($B_{0}>0$), a transformation to a frame rotating with angular frequency $\gamma B_{0}$ removes the static field term $-\gamma B_{0}S^{z}$ from the transformed Hamiltonian at the cost of introducing time-dependent, sinusoidal terms in the equations of motion. Then, the argument goes, if these sinusoidal terms oscillate sufficiently rapidly, their effect on the motion averages out [6,11]. Although this argument holds for $B_{0}\to \infty$, for realistic values of $B_{0}$ and $B_{1}$, see Section 3.3, it does not. Only if the particle trajectories are close to the region where the field gradient is small, the argument applies, see Appendix A. When applied to the SG experiment with realistic values of $B_{0}$ and $B_{1}$, the above argument is circular but self-consistent. The justification that the argument is valid comes from the numerical solution presented in Section 3.5.

If we simply omit the *x*-component in Equations (7) and (8) (and thereby violate one of Maxwell’s equations), we are left with the classical problem in which $S^{z}$ does not change with time and the particle is subject to a force $F\left(x\right)=\gamma \;B_{1}S^{z}e_{z}$ (recall that $B_{0}$ has disappeared because of the transformation to the rotating frame). For the initial conditions $x=(0,y_{0},0)$ and $v=(0,v_{y},0)$ we have $v_{x}\left(t^{*}\right)=0$, $v_{z}\left(t^{*}\right)=\pm \hbar \gamma B_{1}S^{z}t^{*}/m$, $x(t^{*})=0$, and $z\left(t^{*}\right)=\pm \hbar \gamma B_{1}S^{z}{\left(t^{*}\right)}^{2}/2m$. The expressions for final velocity $v_{z}\left(t^{*}\right)$ and position $z(t^{*})$ are the same as those obtained in the first analytically solvable case. For each random choice of S, $S^{z}$ is a random number in the range [−1/2,1/2] and the distribution of velocities is a line at $v_{x}=0$, stretching from $v_{z}=-v^{*}$ to $v_{z}=v^{*}$. This is the expected outcome of the Newtonian description of the SG experiment that is often referred to when comparing with the quantum-theoretical prediction.

### 3.3. Model Parameters

We adopt the geometry of the experiment with neutrons, reported in Ref. [10]. The region in which there is a nonzero gradient in the *x*-*z* directions is 0.8m long [10], that is $y_{1}-y_{0}=0.8\;m$. In the neutron experiment, the maximum gradient of the *B*-field is estimated to be $B_{1}=300\;T/m$ [10]. In the case of the SG experiment with silver atoms, estimates range from $B_{1}=1\;T/\mathrm{cm}=100\;T/m$ to $B_{1}=20\;T/\mathrm{cm}=2000\;T/m$ [9,27]. In view of the uncertainties about the strength and precise form of the *B*-field gradients in these experiments and taking into consideration that the simple form of the *B*-field gradients used for our theoretical/simulation study is unlikely to hold to any of these experiments, we will use $B_{1}=300\;T/m$ in all our simulation work.

In the case of the experiment with neutrons we have [10,28]

(10) $$\begin{array}{c} \begin{array}{cccccccc} m & = & 1.67\times 10^{-27}\;\mathrm{kg} & , & \gamma & = & -1.83\times 10^{8}\;T^{-1}s^{-1} & ,\\ |\gamma B_{0}| & = & 1.83\times 10^{8}\;s^{-1} & , & |\gamma B_{1}| & = & 5.50\times 10^{10}\;m^{-1}\;s^{-1} & ,\\ \hbar B_{1}/mB_{0} & = & 1.89\times 10^{-5}\;m\;s^{-1} & , & \hbar |\gamma |B_{1}/m & = & 3.46\times 10^{3}\;m\;s^{-2} & ,\\ v_{y} & = & 395.6\;m\;s^{-1} & , & t^{*} & = & 2.02\times 10^{-3}\;s & ,\\ v^{*} & = & 3.50\;m\;s^{-1} & , & z^{*} & = & 3.53\times 10^{-3}\;m & , \end{array} \end{array}$$

where $\hbar =1.05\times 10^{-34}\;\mathrm{kg}\;m^{2}\;s^{-1}$ and we have taken as an example $B_{0}=1\;T$.

Assume, as we did in Section 3.2, that all particles have their initial magnetic moment along the *z*-axis, i.e., S=S(0,0,±1). According to Equation (9), the particles cross the plane at $y=y_{1}$ at $x=\left(0,y_{1},\pm v^{*}t^{*}/2\right)=\left(0,y_{1},\pm 3.53\times 10^{-3}\;m\right)$ with a velocity $v=(0,v_{y},\pm 3.50\;m\;s^{-1})$. During the remaining free-particle flight to the detector screen, the *z*-coordinate changes by $\Delta z_{\mathrm{screen}}=\pm 3.50\times 2\;m/395.6=\pm 17.7\;\mathrm{mm}$. Thus, in traveling from the source to the detector, the *z*-coordinate changes by $\Delta z_{\mathrm{source}-\mathrm{screen}}\approx \pm 21.2\;\mathrm{mm}$. This is about a factor of 7 larger than the splitting observed in the neutron experiment [10]; see Figure 1. In view of the fact that the magnetic field Equation (7) is unlikely to result from the real magnet used in the experiment [10], this order-of-magnitude agreement between the beam-splittings at the screen is quite satisfactory.

As explained in Appendix D, solving the time-dependent Pauli equation for the quantum-theoretical model with the set of parameters given by Equation (10) is computationally very expensive. In order to speed up the development of the simulation software and to generate simulation data for a case that is substantially different than that of neutrons, we have chosen to perform simulations with parameters taken from the original SG experiment [9,27] except that instead of the value of magnetic moment of the silver atom, we have taken the value of the magnetic moment of the Ag${}^{107}$ nucleus [29]. In the following, we refer to this case as simulations with imaginary silver particles. The parameters are

(11) $$\begin{array}{c} \begin{array}{cccccccc} m & = & 1.79\times 10^{-25}\;\mathrm{kg} & , & \gamma & = & -1.09\times 10^{7}\;T^{-1}s^{-1} & ,\\ |\gamma B_{0}| & = & 1.09\times 10^{7}\;s^{-1} & , & |\gamma B_{1}| & = & 3.26\times 10^{9}\;m^{-1}\;s^{-1} & ,\\ \hbar B_{1}/mB_{0} & = & 1.76\times 10^{-7}\;m\;s^{-1} & , & \hbar |\gamma |B_{1}/m & = & 1.91\;m\;s^{-2} & ,\\ v_{y} & = & 540\;m\;s^{-1} & , & t^{*} & = & 1.48\times 10^{-3}\;s & ,\\ v^{*} & = & 1.42\times 10^{-3}\;m\;s^{-1} & , & z^{*} & = & 1.05\times 10^{-6}\;m & , \end{array} \end{array}$$

where again, we have taken as an example $B_{0}=1\;T$.

### 3.4. Numerical Solution of Equation (3)

In practice, we solve the system Equation (3) by a combination of the exact integration of the torque equation Equation (5) and the velocity-Verlet method as used in molecular dynamics [30]. Appendix B gives the details of the algorithm that we use.

Unless mentioned explicitly, the model parameters for all our classical simulations are $B_{1}=300\;T/m$. Numerical experiments show that the simulation results show insignificant quantitative changes if we decrease the time step from $\tau =10^{-8}\;s$ to $\tau =10^{-9}\;s$. We use the latter to compute the data that we present in this paper.

Solving Equation (3) for N= 1,000,000 particles with a time step of $\tau =10^{-9}\;s$ takes of the order of hundred minutes on a compute node with two 24-cores Intel Xeon Platinum 8168 CPUs running at 2.7 GHz. We only present data that are essential for the comparison of the classical and quantum description of an SG experiment.

### 3.5. Newtonian Dynamics: Simulation Results for Neutrons

**Figure 3:**
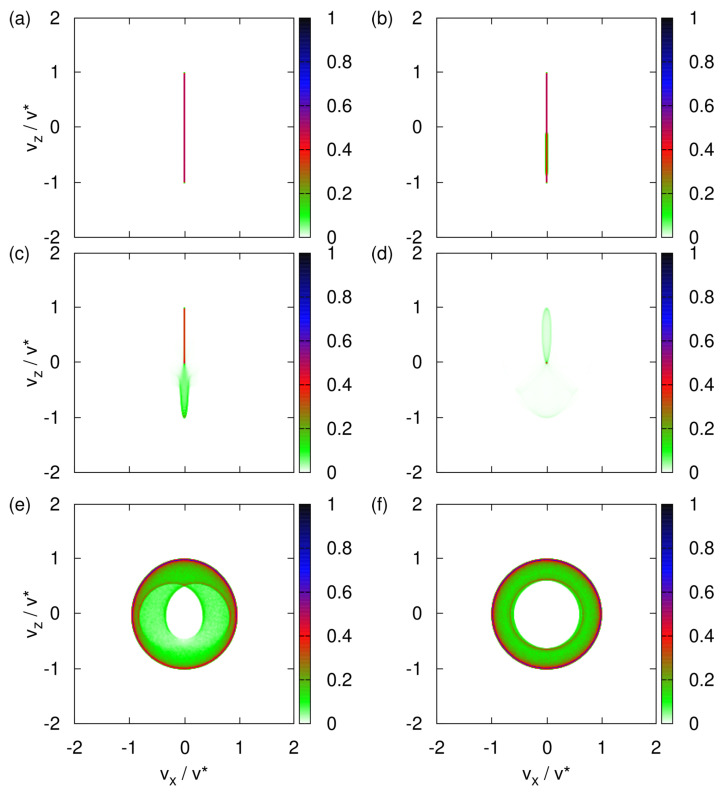
Histograms of the transverse velocity distribution obtained by the solving the classical equations of motion Equation (3) with the initial magnetic moments distributed randomly (see text) and for different values of the uniform magnetic field $B_{0}$. (**a**) $B_{0}=1\;T$; (**b**) $B_{0}=0.1\;T$; (**c**) $B_{0}=0.01\;T$; (**d**) $B_{0}=0.001\;T$, hard to see but looks similar to a projection of an elongated pacifier; (**e**) $B_{0}=0.0001\;T$; (**f**) $B_{0}=0.00001\;T$.

In this section, we focus on the SG with neutrons [10]. Repeating the simulations with the particle parameters of imaginary silver particles (see Equation (11) yields data, some of which are presented in Figure 3 and Appendix C, that leads to the same general conclusions.

To allow for a spreading of the particle beam entering the magnet, the distance from the source to the magnet $y_{0}=1\;m$, similar to the distance between the rightmost collimator and the magnet in the neutron experiment [10]. The length of the magnet in the *y*-direction $y_{1}-y_{0}=0.8\;m$ [10]. The distance from the magnet to the detection screen is taken to be zero because the motion of the particle is that of a free particle with velocity $v=(\Delta v_{x},v_{y},\Delta v_{z})$ where $\Delta v_{x}$ and $\Delta v_{z}$ are the changes of the transverse velocities due to the magnetic field gradients.

As the particles leave the source, the positions and velocities are normally distributed, centered around x=(0,0,0) and $v=(0,v_{y},0)$ and with variances $\sigma_{x}$ and $\sigma_{v}$, respectively. Unless mentioned explicitly, $\sigma_{x}=\sigma_{v}=0$.

The simulation reproduces the analytically obtained results if the initial positions and velocities are $x=(0,y_{0},0)$ and $v=(0,v_{y},0)$, respectively, and the initial magnetic moments are aligned along the *z*-axis, i.e., S=(0,0,±1)/2. The results are in excellent agreement with those obtained by solving the problem analytically and are, therefore, not shown.

Next, we assume that the direction of the magnetic moment, represented by the three-dimensional spin vector S, is uniformly distributed over the sphere. In different words, there is maximum uncertainty about the directions of magnetic moments of the neutrons emerging from the neutron guide (see Figure 1). With this initial condition of S, the transverse velocity distribution changes drastically as the strength of the uniform magnetic field decreases from rather strong ($B_{0}=1\;T$) to very weak ($B_{0}\approx 0\;T$), as illustrated in Figure 3a–f.

From Figure 3a, it follows that a uniform magnetic field of $B_{0}=1\;T$ is sufficiently strong to suppress the effect of the *x*-component of the magnetic field. Because the spins S of different particles are distributed uniformly over the sphere of radius S=1/2, the final distribution of velocities is a strip at $v_{x}\approx 0$, stretching from $v_{z}=-v^{*}$ to $v_{z}=v^{*}$. This is exactly as expected [1,3,4,5,6,7] on the basis of the arguments discussed in Section 3.2.

Figure 3b–e demonstrate that the transverse velocity distribution changes drastically each time we reduce $B_{0}$ by an order of magnitude. Analytically predicting any of particular shapes shown in Figure 3c–e seems to be a daunting task.

For $B_{0}=0$, any rotation of the (x,z) coordinates about the *y*-axis together with the corresponding inverse rotation of the spin leaves the Hamiltonian invariant. As the initial values of $\left(S^{x},S^{z}\right)$ are distributed uniformly over a circle it follows that the maxima of the transverse velocity distribution are expected to trace out a circle in the $v_{x}$-$v_{z}$ plane, in agreement with Figure 3f.

**Figure 4:**
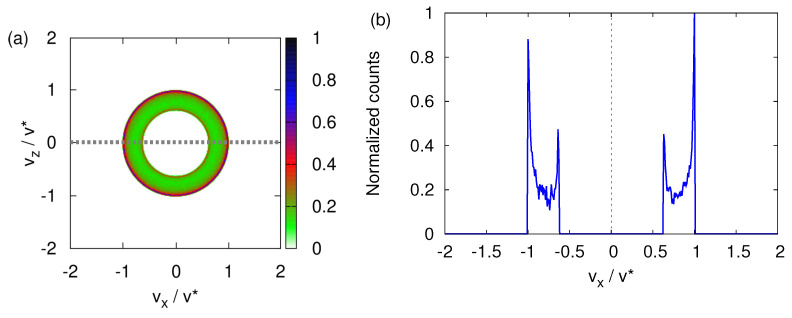
(**a**) Histogram of the transverse velocity distribution obtained by solving the classical equations of motion Equation (3) for $B_{0}=0$, with the initial magnetic moments distributed randomly (see text). (**b**) Distribution of the number of particles obtained by integrating the histogram shown in (**a**) for $v_{z}\in \left[-v^{*},v^{*}\right]/100$, as indicated by the gray dashed line in (**a**).

Figure 4 shows data for the case $B_{0}=0$. The transverse velocity distribution looks very similar to the one shown in Figure 3f. In the neutron experiment [10], the neutrons that have passed through the SG magnet are selected by means of a narrow window that moves in one direction (say the *x* direction) only. The recorded neutron counts, plotted as a function of *x*, show two, very well-separated maxima (see Figure 2). In analogy with the experimental procedure, we compute the one-dimensional, *x*-dependent distribution by integrating the histogram shown in Figure 4a for $v_{z}\in \left[-v^{*},v^{*}\right]/100$. This procedure is the computational equivalent of the moving window used in the neutron experiment. The resulting *x*-dependent distribution is displayed in Figure 4b. This projected transverse velocity consists of two very well-separated distributions. Figure 4b strongly suggests that the presence of a magnetic field gradient causes the incident beam of particles to split into two well-defined beams.

**Figure 5:**
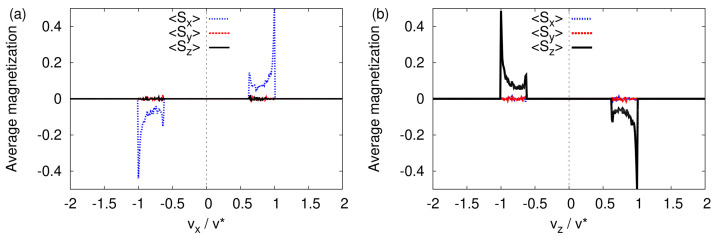
Histogram of the average of the three spin components in the transverse velocity distribution obtained by solving the classical equations of motion Equation (3) with the initial magnetic moments distributed randomly (see text). (**a**) Average calculated by integrating the spin data for $v_{z}\in \left[-v^{*},v^{*}\right]/100$. (**b**) Average calculated by integrating the spin data for $v_{x}\in \left[-v^{*},v^{*}\right]/100$.

In Figure 5, we present the corresponding data for three spin components $S^{x}$, $S^{y}$, and $S^{z}$, obtained by averaging the respective values for $v_{z}\in \left[-v^{*},v^{*}\right]/100$ (Figure 5a) and $v_{x}\in \left[-v^{*},v^{*}\right]/100$ (Figure 5b), respectively. Both figures clearly show that the presence of the magnet field gradient causes the initially randomly oriented spins S to preferably align along the direction of transverse propagation.

More specifically, focusing on the peaks at $v_{z}/v^{*}=\pm 1$ in Figure 5b, we find that the particles with $S^{z}\approx 1/2$ (and $S^{x}\approx 0$, $S_{y}\approx 0$) acquired a negative transverse velocity, whereas those with $S^{z}\approx -1/2$ (and $S^{x}\approx 0$, $S^{y}\approx 0$) acquired a positive transverse velocity, in qualitative agreement with the quantum-theoretical description (see Section 4.1). Similarly, looking at Figure 5a, we conclude that particles with $S^{x}\approx 1/2,-1/2$ (and $S^{y}\approx 0$, $S^{z}\approx 0$) acquired a positive (negative) transverse velocity, also in qualitative agreement with the quantum-theoretical description (see Section 4.1). The fact that for the *x*-direction, positive and negative are interchanged with respect to the case of the *z*-direction is a direct consequence of the different signs of the corresponding components of the magnetic field (see Equation (8)).

Viewed along one direction, e.g., the *z*-direction, there are two well-separated beams, each of which has a well-defined magnetization. Thus, in the absence of the uniform magnetic field ($B_{0}=0$), the classical Newtonian model yields a one-dimensional profile that displays all signatures of the “quantization of the magnetic moment”. Or, put differently, unless the uniform magnetic field $B_{0}$ is sufficiently strong, the classical Newtonian model predicts “quantization of the magnetic moment” in any direction.

**Figure 6:**
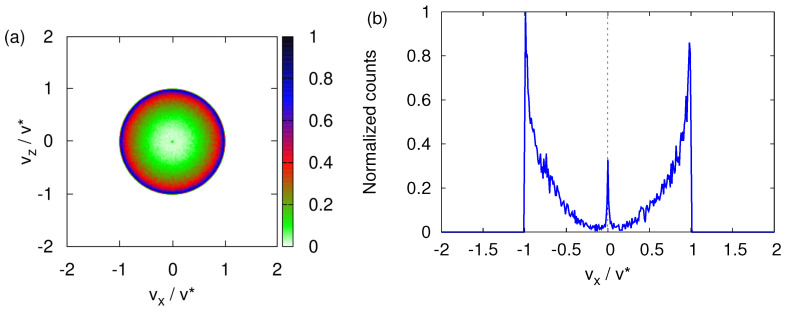
(**a**) Same as Figure 4a except that as the particles depart from the source, the variance of the transverse velocity $\sigma_{v}=0.28v^{*}$. (**b**) Distribution of the number of particles obtained by integrating the histogram shown in (**a**) for $v_{z}\in \left[-v^{*},v^{*}\right]/100$. The distribution obtained by integrating the same histogram for $v_{x}\in \left[-v^{*},v^{*}\right]/100$ looks identical and is therefore not shown.

For completeness, Figure 6 shows how a spread in the initial transverse velocities affects the final transverse velocity distribution for $B_{0}=0$. Clearly, the main features displayed in Figure 4 are prominently present.

**Figure 7:**
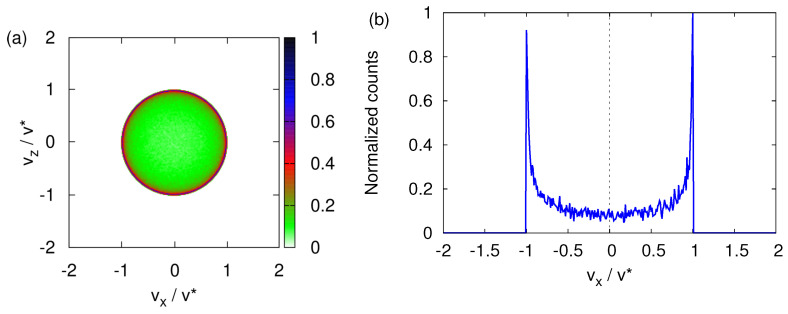
Same as Figure 4 except that the parameters for neutrons have been replaced by the parameters for imaginary silver particles.

Finally, Figure 7 shows that performing the classical simulation using model parameters appropriate for imaginary silver particles instead of neutrons does not change the qualitative features of the transverse velocity distribution. Compared to neutrons (see Figure 4b), the main difference is that the transverse velocity distribution is more spread out over the circle with radius $v^{*}$ (see Figure 7b)

## 4. Quantum-Theoretical Model

The Hamiltonian describing a neutral, spin-1/2 particle of mass *m* subject to a time-independent magnetic field B=B(x) reads

(12) $$\begin{array}{ccc} H & = & \frac{1}{2m}p^{2}-\frac{\hbar \gamma }{2}B\left(x\right)\cdot \sigma \;, \end{array}$$

where $p=(p_{x},p_{y},p_{z})=-i\hbar \nabla$ are the momentum operators, $\sigma =(\sigma^{x},\sigma^{y},\sigma^{z})$ are the three Pauli matrices and γ is the gyromagnetic ratio. The magnetic field B(x) is given by Equation (7).

From Equations (7) and (12), it follows immediately that $[p_{y},H]=0$, that is, the momentum in the *y*-direction is conserved. In other words, the motion of the particles in the *y*-direction is that of a free particle; therefore, in the region where the magnetic field is nonzero, the quantum-theoretical problem effectively amounts to solving the TDPE

(13) $$\begin{array}{ccc} i\frac{\partial }{\partial t}|\Psi \left(t\right)\rangle & = & \left[-\frac{\hbar }{2m}\left(\frac{\partial^{2}}{\partial x^{2}}+\frac{\partial^{2}}{\partial z^{2}}\right)-\frac{\gamma B_{0}}{2}\sigma^{z}-\frac{\gamma B_{1}}{2}z\sigma^{z}+\frac{\gamma B_{1}}{2}x\sigma^{x}\right]|\Psi \left(t\right)\rangle \;, \end{array}$$

for the two-component spinor

(14) $$\begin{array}{c} \langle x,z|\Psi \left(t\right)\rangle =\left(\begin{array}{c} \Psi_{+1}\left(x,z,t\right)\\ \Psi_{-1}\left(x,z,t\right) \end{array}\right)\;, \end{array}$$

where the subscript s=±1 refers to the eigenvalues *s* of the $\sigma^{z}$ operator.

In Appendix D, we discuss the details of the analytical and numerical tools we use to solve Equation (13).

### 4.1. Quantum Theory: Simulation Results

**Figure 8:**
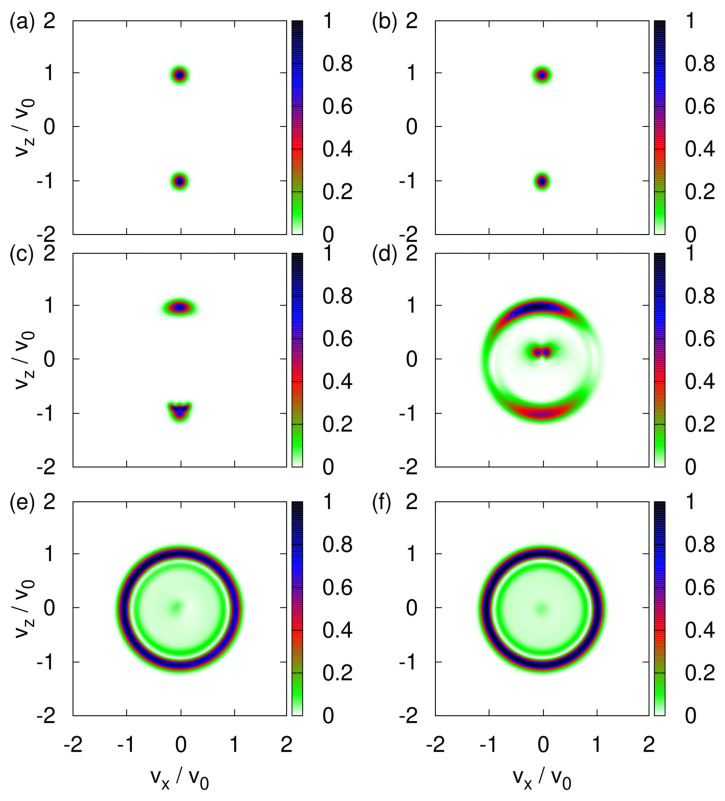
Probability distribution $|\langle v_{x},v_{z}|\Phi \left(t^{*}/10\right)\rangle {|}^{2}$ ($v_{0}=v^{*}/10$) obtained by solving the TDPE Equation (A24) with the initial state given by Equation (A27). Initially, the (dimensionless) variance σ=0.1 and the spin state is $(|↑\rangle +\left|↓\rangle \right)/\sqrt{2}$. (**a**) $B_{0}=1\;T$; (**b**) $B_{0}=0.1\;T$; (**c**) $B_{0}=0.01\;T$; (**d**) $B_{0}=0.001\;T$; (**e**) $B_{0}=0.0001\;T$; (**f**) $B_{0}=0.00001\;T$.

Figure 8 shows the transverse velocity distribution $|\langle x,z|\Phi \left(t^{*}/10\right)\rangle {|}^{2}$ obtained by solving the TDPE Equation (A24) for various strengths of the uniform magnetic field and up to the time $t^{*}/10$ at which, in the Newtonian model, the neutrons would have left the region in which the magnetic field is present.

For a sufficiently strong uniform magnetic field, e.g., $B_{0}=1\;T$, the transverse velocity distribution is bimodal with well-separated maxima at $v_{z}\approx \pm v_{0}$; see Figure 8a. The SG magnet then functions as an (almost perfect) filtering device, yielding particle beams which *may* be labeled by the eigenvalues of the $\sigma^{z}$ Pauli matrix.

We wrote *may* because a meaningful assignment in terms of the eigenvalues $\sigma^{z}$ requires that if we send the beam of particles through a second SG magnet with its strong uniform magnetic field along the *z*-axis, the particles should emerge in one and the same beam only.

More generally, if we use a filter device to label different outcomes, subsequent repeated filtering by identical devices should leave the labeling intact [31]. If it does not, the original assignment is useless.

Thus, to verify that an SG magnet with its strong uniform magnetic field along the *z*-axis acts as a spin-filtering device, we repeat the simulation with $B_{0}=1\;T$ and initial spin state |↑〉. The resulting transverse velocity distribution is the same as the one in Figure 8a with the top spot removed (image not shown). Thus, with a strong static field $B_{0}$, the SG magnet indeed acts as an ideal filtering device.

In the quantum-theoretical treatment, the spin is quantized by construction; therefore, the observed splitting of the beam cannot be regarded as evidence for the quantization of the spin; however, for large $B_{0}$, the quantized spin model shows that the SG magnet splits the beam (in agreement with experiment) whereas the Newtonian model does not (in disagreement with experiment), exposing a fundamental shortcoming of the latter.

As in the classical case (see Figure 3a–f), the transverse velocity distribution changes drastically with each reduction of $B_{0}$ by an order of magnitude; see Figure 8a–f. The distributions for large (Figure 8a,b) and small (Figure 8e,f) values of the uniform magnetic field $B_{0}$ are robust to changes of $B_{0}$ but for intermediate values of $B_{0}$ (Figure 8c,d), it is hard to predict the distribution. The distributions shown in Figure 8e,f look very similar to their classical counterparts shown in Figure 3e,f but differ in the details.

Maxwell’s equation dictates that (with our choice of the frame of reference) the Hamiltonian of an SG experiment should contain terms in both $\gamma \sigma^{x}B_{1}$ and $\gamma \sigma^{z}B_{1}$, which implies that the magnetization (in any direction) is not conserved. Therefore, unless $B_{0}\to \infty$, the eigenvalues of $\sigma^{z}$ cannot be used to label the eigenstates of the Hamiltonian. In other words, there are situations, choices of the model parameters, for which the SG magnet cannot be used to define the quantization direction of the spin [21,22].

**Figure 9:**
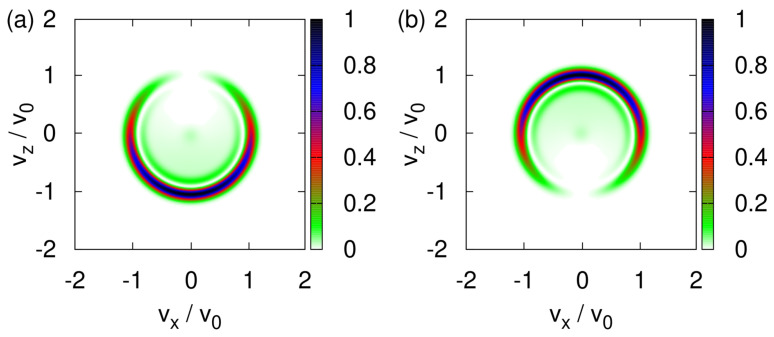
(**a**) Probability distribution $|\langle v_{x},v_{z}|\Phi \left(t^{*}/10\right)\rangle {|}^{2}$ ($v_{0}=v^{*}/10$) of the transverse velocity distribution obtained by solving the TDPE Equation (A24) with the initial state given by Equation (A27) and $B_{0}=0$. Initially, the (dimensionless) variance σ=0.1 and the spin state is |↑〉. (**b**) Same as (a) except that the initial spin state is |↓〉.

We study this aspect by solving the TDPE for the initial state given by Equation (A27) with θ=α=0, that is for the initial spin states |↑〉 and |↓〉 and $B_{0}=0$. The transverse velocity distributions are shown in Figure 9a,b. If the initial spin state is |↑〉 (|↓〉), the wave packet dominantly propagates along the −z-direction and +z-direction; see Figure 9a,b, respectively. The term $\gamma \sigma^{x}B_{1}$ causes both components of the wave function to spread in all directions, producing the sickle-like shapes in Figure 9a,b. Not surprisingly, the sum of Figure 9a,b yields an image that looks very much like Figure 8f.

**Figure 10:**
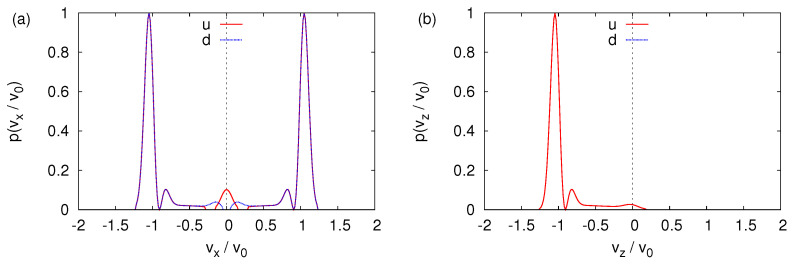
(**a**) One-dimensional probability distributions $p\left(v_{x}\right)={\left|\Phi_{+1}\left(v_{x},v_{z}=0,t^{*}/10\right)\right|}^{2}$ (u, solid line) and $p\left(v_{x}\right)={\left|\Phi_{-1}\left(v_{x},v_{z}=0,t^{*}/10\right)\right|}^{2}$ (d, dotted line), extracted from the data shown in Figure 9a. Except for $|v_{x}/v_{0}|\leq 0.2$, the difference between two distributions is too small to be visible in the plot. (**b**) One-dimensional probability distributions $p\left(v_{z}\right)={\left|\Phi_{+1}\left(v_{x}=0,v_{z},t^{*}/10\right)\right|}^{2}$ (u, solid line) and $p\left(v_{z}\right)={\left|\Phi_{-1}\left(v_{x}=0,v_{z},t^{*}/10\right)\right|}^{2}$ (d, dotted line), extracted from the data shown in Figure 9a. The probability distributions $|\Phi_{-1}\left(v_{x}=0,v_{z},t^{*}/10\right){|}^{2}$ is too small to be visible in the plot. Except for $|v_{x}/v_{0}|\leq 0.2$, the difference between two distributions is too small to be visible in the plot. For presentation purposes, each distribution is normalized such that its maximum is one. As in Figure 9, $v_{0}=v^{*}/10$.

Figure 10a,b shows the corresponding probability distributions for the |↑〉 and |↓〉 components of the wave function, projected onto the z=0 and x=0 axis, respectively.

If, in an experiment such as the one with cold neutrons [10], one would only count particles by moving a narrow window along the *x*-direction, the distribution shown in Figure 10a would lead us to conclude that the SG magnet has split the beam into parts. On the other hand, measuring with a moving window along the *z*-direction yields the distribution shown in Figure 10b, which forces us to conclude that only the |↓〉 component is present in the outgoing beam. Indeed, the intensity of the |↑〉 component is several orders of magnitude smaller than the one of the |↓〉 component. For $B_{0}≳0$, the SG magnet does not act as a spin filter.

It may be of interest to note that if an SG magnet is used to measure the magnetic moment to, e.g., atomic clusters [13], the value of $B_{0}$ does not matter much. The positions of the peaks in the one-dimensional distributions, which are the same for large and zero uniform magnetic field $B_{0}$, suffice to determine the value of the magnetic moment.

### 4.2. Quantum Theory: Simplified Model

The TDPE Equation (13), with the term in $\sigma^{x}$ removed, is an excellent approximation to the full TDPE Equation (13) if the uniform magnetic field is strong enough, e.g., if $B_{0}=1\;T$. Then, we may also replace $\sigma^{z}$ by σ·b where b=B/∥B∥ is the unit vector parallel to the strong, uniform magnetic field, because (i) the eigenvalues of σ·b are the same as those of $\sigma^{z}$ and (ii) only the eigenvalues enter in the spin part of the simplified TDPE. By introducing b, the latter can describe situations in which the strong uniform magnetic field can take any orientation, as long as it is approximately perpendicular to the *y*-direction (otherwise the argument to remove the $\sigma^{x}$ term may break down).

We can now simplify the description further. Because of the one-to-one correspondence between the eigenvalue of σ·b and the change in the transverse velocity of the outgoing particles, we may dispose of the description of the translational degrees of freedom entirely and represent the operation of the SG apparatus by the projection operator [7]

(15) $$\begin{array}{ccc} M(b) & = & \frac{1+\sigma \cdot b}{2}\;, \end{array}$$

acting on the spin state $|\psi \rangle =a_{↑}|↑\rangle +a_{↓}|↓\rangle$ only. The probability to observe a particle in the beam labeled by $s_{b}=\pm 1$, one of the two eigenvalues of b·σ, is given by

(16) $$\begin{array}{ccc} P(s_{b}|\xi ) & = & \langle \psi |M\left(b\right)|\psi \rangle =\frac{1+s_{b}\;\cos\xi }{2}=\left\{\begin{array}{ccc} \cos^{2}\frac{\xi }{2} & , & s_{b}=+1\\ \sin^{2}\frac{\xi }{2} & , & s_{b}=-1 \end{array}\right.\;, \end{array}$$

where cosξ=s·b and s=〈ψ|σ|ψ〉. The last expression in Equation (16) is reminiscent of Malus’ law for the intensity of polarized light passing through a polarizer.

The projector equation (Equation (15)) and the probability equation (Equation (16)) describe the operation of the SG apparatus in terms of the spin-degree of freedom only. This simplified model is often used in textbooks to elucidate quantum measurement theory [5,6,7]. We stress that Equation (16) does not apply to the case of a weak uniform magnetic field.

Solving the TDPE Equation (13) for $B_{0}=1\;T$ and for the initial states Equation (A27) with θ=0,π/6,π/4,π/3 and α=θ/2 yields the expected bimodal shape of the transverse velocity distributions (data not shown). The total probabilities for $v_{z}<0$ and $v_{z}>0$ are in excellent agreement with the prediction based on Equation (16).

## 5. Event-by-Event Simulation

**Figure A1:**
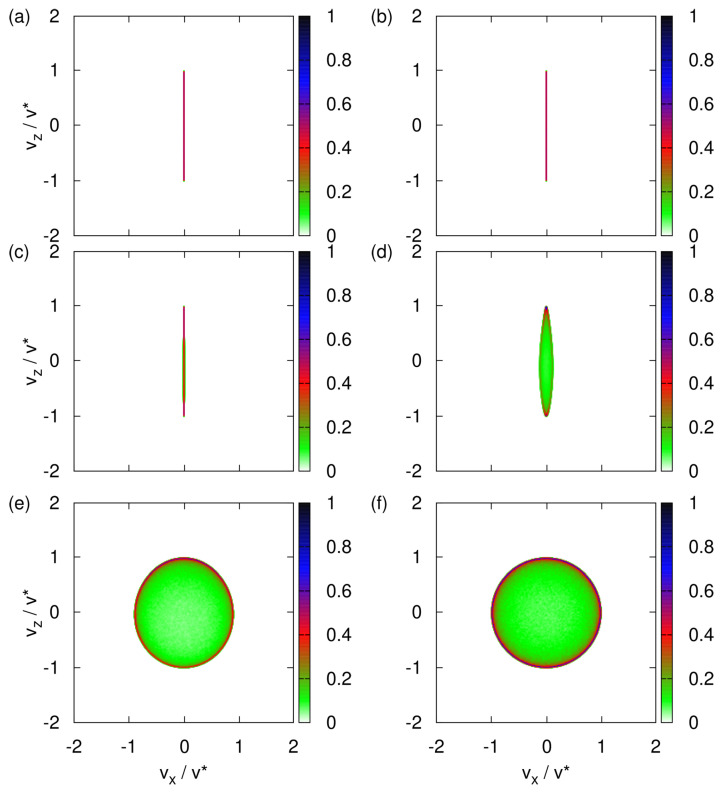
Histograms of the transverse velocity distribution obtained by solving the classical equations of motion Equation (3) for the model parameters pertaining to imaginary silver particles and for different values of the uniform magnetic field $B_{0}$. The initial magnetic moments distributed randomly (see text) (**a**) $B_{0}=1\;T$; (**b**) $B_{0}=0.1\;T$; (**c**) $B_{0}=0.01\;T$; (**d**) $B_{0}=0.001\;T$; (**e**) $B_{0}=0.0001\;T$; (**f**) $B_{0}=0.00001\;T$.

**Figure A2:**
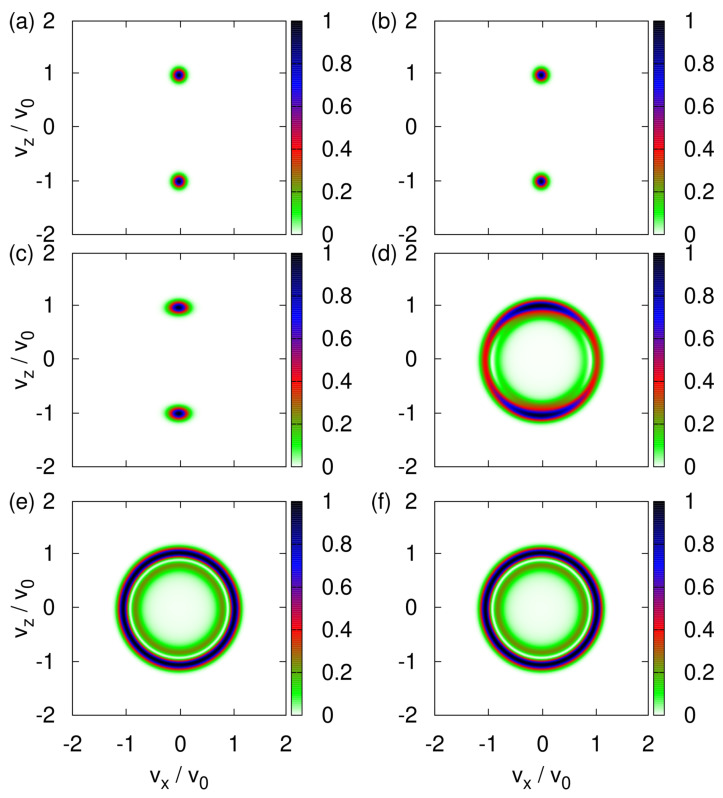
Probability distributions $|\langle v_{x},v_{z}|\Phi \left(t^{*}\right)\rangle {|}^{2}$ of the transverse velocity distribution obtained by solving the TDPE Equation (A24) with the initial state given by Equation (A27) and for the model parameters pertaining to imaginary silver particles ($v_{0}=v^{*}$). Initially, the variance (dimensionless) σ=0.1 and the spin state is $(|↑\rangle +\left|↓\rangle \right)/\sqrt{2}$. (**a**) $B_{0}=1\;T$; (**b**) $B_{0}=0.1\;T$; (**c**) $B_{0}=0.01\;T$; (**d**) $B_{0}=0.001\;T$; (**e**) $B_{0}=0.0001\;T$; (**f**) $B_{0}=0.00001\;T$.

From the comparison of Figure 3a with Figure 8a and also of Figure A1a with Figure A2b, it is immediately clear that the transverse velocity distributions are very different if $B_{0}=1\;T$. For $B_{0}=0$, there is no qualitative difference between the Newtonian and quantum-theoretical results.

The qualitative difference between the Newtonian and quantum-theoretical prediction in the case of a large uniform magnetic field has been decisive to eliminate the former as a description of the experimental observations [1,2,3]; however, that does not imply that quantum theory is the only viable description of experiments in which the frequency distribution of detection events is built up one-by-one, such as in the SG experiment.

From this broader perspective, the fundamental question to be answered is “is it possible to construct a process that generates event-by-event and without using knowledge about the final distribution of events, frequency distributions that are commonly thought to be a signature of wave interference, two-particle entanglement, uncertainty, etc.” This question is answered in the affirmative by the event-by-event simulation approach developed in Refs. [32,33,34,35,36,37,38,39,40,41,42,43].

In the case at hand, the conceptually interesting question is whether it is possible to retain a picture of the SG experiment in which individual particles follow trajectories while, in contrast to the Newtonian results, the transverse velocity distribution exhibits two well-separated maxima along the line defined by the direction of the strong static field (the *z* direction in our case).

Remarkably, a marginal modification of Newton’s equation of motion suffices to answer this question affirmatively. The modification consists of replacing step

4. If $y\in [y_{0},y_{1}]$ set $F=\gamma \;B_{1}S^{z}e_{z}-\gamma \;B_{1}S^{x}e_{x}$,

in which the force F is being calculated (see Appendix B for details) by the rules

4. If $y\in [y_{0},y_{1}]$: the **first** time that the event ∥B(x)∥>0 occurs, that is when the particle enters the region where ∥B(x)∥>0, use Equation (16) with s=S to align the vector S along the magnetic field $s_{b}B\left(x\right)$ and compute $F=\gamma \;B_{1}S^{z}e_{z}-\gamma \;B_{1}S^{x}e_{x}$.

In detail, if r≤S·B(x)/∥B∥ set S=B/2∥B∥, otherwise set S=−B/2∥B∥. Here *r* is a uniform (pseudo) random number in the range [−1/2,1/2] (which changes each time before it is used). With the new S, compute $F=\gamma \;B_{1}S^{z}e_{z}-\gamma \;B_{1}S^{x}e_{x}$. For each particle, the alignment of S is carried out only once.

One might try to argue that because the event-by-event model makes use of Equation (16), it implicitly “knows” about quantum theory; however, probabilistic laws such as Equation (16) also follow from the application of logical-inference [19,44] to the modeling of event-based processes. This approach yields Equation (16) directly, without any reference to quantum-theoretical concepts.

In short, the key idea of the logical inference approach is that “good” physics experiments must yield reproducible frequency distributions which are robust, meaning do not change much, if the conditions under which the data was taken changes a little [44]. In the case at hand, the frequency distribution consists of the average numbers of +1 and −1 events and ξ=arccos(2S·B(x)/∥B∥) represents the condition [44]. Expressing the key idea mathematically leads to the requirement that the Fisher information

(17) $$\begin{array}{ccc} I_{F}\left(\xi \right) & = & \sum_{x=\pm 1}\frac{1}{p(x|\xi )}{\left(\frac{\partial p(x|\xi )}{\partial \xi }\right)}^{2}>0\;, \end{array}$$

for the probability p(x|ξ) to observe the event x=±1 under the condition ξ must be independent of ξ and minimal [44]. After some elementary algebra, we find that the solution of this optimization problem reads [44]

(18) $$\begin{array}{ccc} p(x|\xi ) & = & \frac{1\pm x\cos\xi }{2}\;, \end{array}$$

where the ± sign reflects the ambiguity in assigning +1 or −1 to one of the directions. Quantum theory postulates Equation (16) (through the Born rule) whereas the logical inference approach allows us to derive Equation (16) without making reference to a concept of quantum theory. Thus, the argument that the event-by-event algorithm implicitly refers to quantum theory does not hold.

Moreover, the modification does not change the vector character of S. In the event-by-event model, S can take any value on the sphere of radius 1/2, there is no wave function, there are no Pauli spin matrices, there simply is no element of quantum theory in the event-by-event model.

**Figure 11:**
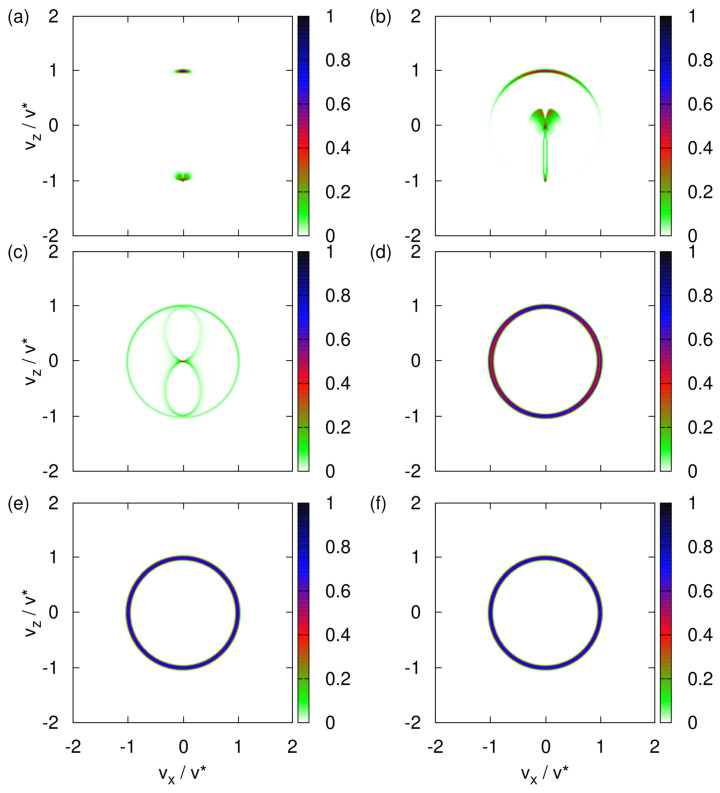
Histograms of the transverse velocity distribution obtained by event-by-event simulation. The classical equations of motion (Equation (3)) are modified to include a one-time projection of the spin vector S along the direction of the magnetic field using the procedure described in the text. The initial magnetic moments of the particles are distributed randomly. The variance of the transverse velocity $\sigma_{v}=0.014v^{*}$. (**a**) $B_{0}=1\;T$; (**b**) $B_{0}=0.1\;T$; (**c**) $B_{0}=0.01\;T$; (**d**) $B_{0}=0.001\;T$; (**e**) $B_{0}=0.0001\;T$; (**f**) $B_{0}=0.00001\;T$.

Figure 11a demonstrates that the event-based model produces a bimodal transverse velocity distribution, in qualitative agreement with the solution of the TDPE Equation (13). Clearly, the minor modification to Newton’s equation has a tremendous impact on the trajectories of the particles. For $B_{0}\approx 0$, the event-by-event simulations yields the circular distribution; see Figure 11e,f, in qualitative agreement with both the Newtonian and quantum-theoretical description.

As a further check, we perform event-by-event simulations for $B_{0}=1\;T$ and take as initial spin vector $S={\left(\cos\phi \sin\theta ,\sin\phi \sin\theta ,\cos\theta \right)}^{T}/2$ for θ=0,π/6,π/4,π/3, π/2, 2π/3, 3π/4, 5π/6, π and ϕ uniformly random from the interval [0,2π] (data not shown). The total probabilities for $v_{z}<0$ and $v_{z}>0$ are in excellent agreement with Equation (16), that is with quantum theory.

## 6. Conclusions

In all our simulations, the strength of the magnetic field gradient was fixed and tuned to the case of an SG experiment with cold neutrons [10] while the strength of the uniform component of the magnetic field was varied. The simulation data for imaginary silver particles instead of neutrons show the same qualitative features.

In Table 1, we collect the most essential features of the results for the transverse velocity distribution obtained from the simulation of three different models of the SG experiment. Thereby, we have omitted many of the computational details mentioned earlier and limit the discussion to the two extreme cases of a strong and zero uniform magnetic field.

The first three rows of the last column in Table 1 express what is known since the original SG experiment was performed, namely that Newtonian mechanics cannot explain the observed splitting of the particle beam [1,3,4,5,6,7] if the uniform magnetic field component is sufficiently large. It is exactly under this last condition that the quantum-theoretical textbook model provides an accurate description of the time evolution of the probability distribution while the Newtonian model does not.

However, we have also shown that a minor modification to Newton’s equations of motion yields results that are in line with the experimental observation and quantum theory. In this event-by-event simulation approach, the spin is described in terms of a three-dimensional vector, not in terms of Pauli matrices.

If the strength of the uniform magnetic field $B_{0}$ gradually decreases, then, for any of the three models, the changes in the transverse velocity distribution become hard to predict analytically, unless the effect of $B_{0}$ becomes negligible. Indeed, for $B_{0}=0$ a symmetry argument can be used to understand why the calculated transverse velocity distribution shows a circular structure, see column two of Table 1.

However, also the case $B_{0}≳0$ poses some interesting interpretational issues, depending on how the distribution of particles is measured. If, as in the neutron experiment [10], one only records the distribution along a particular direction, the Newtonian model also yields a bimodal distribution. Without additional data, the bimodality would (erroneously, see Section 4.1) imply that the two beams can be labeled by the spin quantum number.

From a general perspective, quantization (not to be confused with results from quantum theory) is the process of classifying empirical data into groups and attaching discrete labels to these groups. As mentioned at the end of Section 2, in the specific case of the neutron experiment it is clear that quantization is the result of classification, putting data points in two groups; see Figure 2. Once this “operation” has been carried out, the compressed, new data are “quantized”. In our view, quantum theory provides a powerful mathematical framework to describe such “quantized data”. Within quantum theory, the spin is quantized by definition/construction. If the “quantized” form of the empirical data is described well in terms of a quantum spin model, then that is a great achievement; however, this success does not necessarily justify the conclusion that “quantization” is a property/attribute of the phenomenon that gave rise to the empirical data. In our view, drawing this conclusion mixes up the phenomenon that gave rise to the empirical data with a quantum model of it.

On the basis of SG experiments that have been performed to date, it is not possible to distinguish between the quantum-theoretical and event-by-event model. New, high-precision experiments are needed to rule out the latter and to allow for a quantitative comparison between experimental and simulation data.

Furthermore, it would be of interest to perform an SG experiment in which the uniform magnetic field is weak enough to render the description textbook model invalid. For instance, an experiment with neutrons passing through a quadrupole magnet with a large field gradient would allow a direct comparison with our simulations (which, if needed, can easily be adapted to other field configurations).

## Figures and Tables

**Table 1:** Overview of the shapes of the transverse velocity distributions obtained by computer simulation of three different descriptions of the SG experiment with cold neutrons.

	$B_{0}=0$	$B_{0}=1\;T$
Experiment	???	two spots (Figure 2)
Newton	circular (Figure 3f)	one stripe (Figure 3a)
Quantum theory	circular (Figure 8f)	two spots (Figure 8a)
Event-by-event	circular (Figure 11f)	two spots (Figure 11a)

## Data Availability

The datasets generated during and/or analyzed during the current study are available from the corresponding author on reasonable request.

## Appendix A. Large Static Field *B*_0_

In the following, we assume that the particle is inside the region where the magnetic field $B={\left(-xB_{1},0,\gamma B_{0}+zB_{1}\right)}^{T}$ is present. Written out explicitly, the torque equation for the spin reads

(A1a) $$\begin{array}{ccc} \frac{dS^{x}\left(t\right)}{dt} & = & -\gamma \left(B_{0}+zB_{1}\right)S^{y}\left(t\right)\;, \end{array}$$

(A1b) $$\begin{array}{ccc} \frac{dS^{y}\left(t\right)}{dt} & = & \gamma \left(B_{0}+zB_{1}\right)S^{x}\left(t\right)+\gamma xB_{1}S^{z}\left(t\right)\;, \end{array}$$

(A1c) $$\begin{array}{ccc} \frac{dS^{z}\left(t\right)}{dt} & = & -\gamma xB_{1}S^{x}\left(t\right)\;. \end{array}$$

Next, we only consider classical particles that follow trajectories for which $|x\left(t\right)B_{1}|\ll B_{0}$ and $|z\left(t\right)B_{1}|\ll B_{0}$. For example, if, as in our simulations, we choose $B_{0}=1\;T$ and $B_{1}=300\;T/m$, we require trajectories that satisfy |x(t)|≪3mm and |z(t)|≪3mm which, from the neutron experiment point of view is not unreasonable. Under these conditions, we may ignore the terms in $B_{1}$ in Equations (A1a) and (A1b) and we obtain

(A2) $$\begin{array}{ccc} S^{x}\left(t\right) & = & S^{x}\left(0\right)\cos\gamma B_{0}t-S^{y}\left(0\right)\sin\gamma B_{0}t\;. \end{array}$$

Substituting Equation (A2) into the equation of motion

(A3) $$\begin{array}{ccc} \frac{dv_{x}\left(t\right)}{dt} & = & -\frac{\hbar \gamma B_{1}}{m}S^{x}\left(t\right)\;, \end{array}$$

and integrating over time gives

(A4) $$v_{x}\left(t\right)=v_{x}\left(0\right)+\beta S^{x}\left(0\right)\sin\gamma B_{0}t-\beta S^{y}\left(0\right)\left(1-\cos\gamma B_{0}t\right)\;,$$

where $\beta =\hbar B_{1}/mB_{0}$. Integrating Equation (A4) over time once more results in

(A5) $$\begin{array}{ccc} x(t) & = & x\left(0\right)+v_{x}\left(0\right)t-\beta S^{y}\left(0\right)t+\beta^{\prime }S^{x}\left(0\right)\left(1-\cos\gamma B_{0}t\right)+\beta^{\prime }S^{y}\left(0\right)\sin\gamma B_{0}t\;, \end{array}$$

where $\beta^{\prime }=\beta /\gamma B_{0}$.

In the case of neutrons and for $B_{0}=1\;T$ and $B_{1}=300\;T/m$ we have $\beta \approx 2\times 10^{-5}\;m/s$ and $\beta^{\prime }\approx 10^{-13}\;m$ and it follows immediately from Equation (A5) that the motion of the spin has a negligible effect on the motion of the particles in the *x*-direction.

On the other hand, under the same conditions, it follows from Equations (A1c) and (A2) that $S^{z}\left(t\right)\approx S^{z}\left(0\right)$ and the equation of motion for the *z*-component of the velocity becomes

(A6) $$\begin{array}{ccc} \frac{dv_{z}\left(t\right)}{dt} & = & \frac{\hbar \gamma B_{1}}{m}S^{z}\left(0\right)\;, \end{array}$$

yielding

(A7) $$\begin{array}{ccc} v_{z}\left(t\right) & = & v_{z}\left(0\right)+\frac{\hbar \gamma B_{1}}{m}S^{z}\left(0\right)t\;. \end{array}$$

In our simulations, $S^{z}\left(0\right)$ is a uniform random number in the range [−1/2,+1/2]. Therefore, if $v_{z}\left(0\right)=0$, the values of $v_{z}\left(t^{*}\right)$ are also uniformly distributed over the interval $\left[-v^{*},+v^{*}\right]$.

Clearly, these elementary calculations yield results which are in excellent agreement with the simulation data for $B_{0}=1\;T$.

## Appendix B. Numerical Solution of Equation (3)

For any time step τ, Equation (5) can be solved in closed form. In terms of the three components of the spin vector S, we have

(A8) $$S\left(t+\tau \right)=\left(\begin{array}{c} S^{x}\left(t+\tau \right)\\ S_{y}\left(t+\tau \right)\\ S^{z}\left(t+\tau \right) \end{array}\right)=R\left(\tau \right)\left(\begin{array}{c} S^{x}\left(t\right)\\ S_{y}\left(t\right)\\ S^{z}\left(t\right) \end{array}\right)=R\left(\tau \right)S\left(t\right)\;,$$

where

(A9) $$R\left(\tau \right)=\left(\begin{array}{ccc} u^{2}+v^{2}C+w^{2}C & uv-uvC+wS & uw-uwC-vS\\ uv-uvC-wS & v^{2}+u^{2}C+w^{2}C & vw-vwC+uS\\ uw-uwC+vS & vw-vwC-uS & w^{2}+u^{2}C+v^{2}C \end{array}\right)\;,$$

where $u=\gamma B_{x}/\Omega$, $v=\gamma B_{y}/\Omega$ and $w=\gamma B_{z}/\Omega$, C=cos(τΩ), S=sin(τΩ) and $\Omega =\left|\gamma \right|{\left(B_{x}^{2}+B_{y}^{2}+B_{z}^{2}\right)}^{1/2}$. The matrix R(τ) is orthogonal, implying that the integration scheme does not change the length of S.

We integrate the equations of motion Equation (3) using the velocity-Verlet algorithm [30]. Initially, the positions and velocities are normally distributed, centered around x=(0,0,0) and $v=(0,v_{y},0)$ and with variances $\sigma^{x}$ and $\sigma^{v}$, respectively.

We only consider the case $0<y_{0}<y_{1}$ and $y_{0}\leq y\leq y_{1}$. According to Equation (8), at t=0, the force F(x,t=0)=0. We choose a time step τ and repeat steps 1 to 5:

1. v←v+τF/2m,
2. x←x+τv,
3. S←R(τ)S
4. $F=\gamma \;B_{1}S^{z}e_{z}-\gamma \;B_{1}S^{x}e_{x}$,
5. v←v+τF/2m,

for a number of time steps *N*.

As a curiosity, it may be of interest to mention that if the force F is constant, the Verlet scheme integrates the equation of motions *exactly*. On the other hand, Equations (A8) and (A9) integrate the torque equation for spin *exactly*. Thus, it is only the combination of particle and spin motion that forces us to integrate Equation (3) numerically.

## Appendix C. Newtonian Dynamics: Imaginary Silver Particles

Figure A1c demonstrates that a uniform magnetic field of $B_{0}=0.01\;T$ is sufficiently large to yield a distribution of velocities centered at $v_{x}=0$ and stretching from $v_{z}=-v^{*}$ to $v_{z}=v^{*}$, as expected for the classical, textbook model [1,4,5,6,7].

The transverse velocity distribution changes drastically if $B_{0}$ decreases two and three orders of magnitude; see Figure 3d,e.

As $B_{0}$ vanishes, the transverse velocity distribution becomes a circular disk of radius $v^{*}$, with the maximum located at the edges and the minimum at the center, qualitatively similar to the case of neutrons.

## Appendix D. Quantum-Theoretical Model

If we drop the term in $\sigma^{x}$ in Equation (13), the solution of the corresponding TDPE can be written as a product of unitary operators, each of which can be worked out analytically. Writing $\hat{H}=\tilde{a}\left(p_{x}^{2}+p_{z}^{2}\right)-{\tilde{b}}_{0}\sigma^{z}+{\tilde{b}}_{1}z\sigma^{z}$, we have

(A10) $$\begin{array}{ccc} U(t) & = & e^{-it\hat{H}}=e^{it{\tilde{b}}_{0}\sigma^{z}}e^{-it\tilde{a}p_{x}^{2}}e^{-it\tilde{a}{\left(p_{z}-{\tilde{b}}_{1}t\sigma^{z}/2\right)}^{2}}e^{it{\tilde{b}}_{1}z\sigma^{z}}e^{-i\tilde{a}{\tilde{b}}^{2}t^{3}/12}\;. \end{array}$$

Computing the derivative of U(t) with respect to *t*, it readily follows that U(t) satisfies $\partial_{t}U\left(t\right)=\hat{H}U\left(t\right)$. In other words, if we drop the term in $\sigma^{x}$ in Equation (13), the time-evolution operator of the corresponding TDPE can be solved analytically; see also Appendix D.2 for a more direct proof of this fact.

### Appendix D.1. Momentum Representation

For the particular choice of the magnetic field given by Equation (7), it is advantageous for theoretical and numerical work to write the TDPE Equation (13) in the momentum representation [7]. We define Fourier transform pairs by

(A11) $$\begin{array}{ccc} \Psi (x,z,t) & = & \frac{1}{4\pi^{2}}\int_{-\infty }^{+\infty }\int_{-\infty }^{+\infty }e^{i(k_{x}x+k_{z}z)}\;\Phi \left(k_{x},k_{z},t\right)\;dk_{x}\;dk_{z}\;,\\ \Phi (k_{x},k_{z},t) & = & \int_{-\infty }^{+\infty }\int_{-\infty }^{+\infty }e^{-i(k_{x}x+k_{z}z)}\;\Psi \left(x,z,t\right)\;dx\;dz\;, \end{array}$$

and introduce the operators $q_{x}=i\partial_{k_{x}}$ and $q_{z}=i\partial_{k_{z}}$. Note that $\left[q_{x},k_{x}\right]=\left[q_{z},k_{z}\right]=i$ and that the “*i*” in the definitions of $q_{x}$ and $q_{z}$ is not in the denominator as in the case of the momentum operator in the coordinate representation. The transformation to the momentum representation amounts to making the replacements $x↔q_{x}$, $z↔q_{z}$, $p_{x}↔k_{x}$, $p_{z}↔k_{z}$. In the momentum representation, the TDPE Equation (13) reads

(A12) $$\begin{array}{c} i\frac{\partial }{\partial t}|\Phi \left(t\right)\rangle =\left[\frac{\hbar }{2m}\left(k_{x}^{2}+k_{z}^{2}\right)-\frac{\gamma \;B_{0}}{2}\sigma^{z}-\frac{i\gamma \;B_{1}}{2}\sigma^{z}\frac{\partial }{\partial k_{z}}+\frac{i\gamma \;B_{1}}{2}\sigma^{x}\frac{\partial }{\partial k_{x}}\right]|\Phi \left(t\right)\rangle \;, \end{array}$$

where

(A13) $$\begin{array}{c} \langle k_{x},k_{z}|\Phi \left(t\right)\rangle =\left(\begin{array}{c} \Phi_{+1}\left(k_{x},k_{z},t\right)\\ \Phi_{-1}\left(k_{x},k_{z},t\right) \end{array}\right)\;, \end{array}$$

is the two-component spinor in the momentum representation.

### Appendix D.2. Textbook Model

The TDPE Equation (A12) can be solved in closed form if we ignore the term in Equation (A12) which is proportional to $\sigma^{x}$. Then, the momentum $k_{x}$ and the projection of the spin in the *z*-direction are conserved; therefore, the motion in the *x*-direction is that of a free particle, which we may omit from further considerations. This simplified model is often used to discuss the qualitative aspects of the SG experiment [4,5,6,7,26,45], but it does not comply with the Maxwell equations; however, as explained above, if the uniform magnetic field $B_{0}$ is sufficiently large, the textbook model is an excellent approximation.

Not only is it instructive to derive the closed-form solution of the TDPE of this simplified model, the solution itself is also of great help to validate simulation code. Technically and for consistency, the treatment given below uses the momentum representation (the treatment of which we have not found in the literature).

The TDPE Equation (A12) of the textbook model separates into two uncoupled first-order partial differential equations

(A14) $$\begin{array}{ccc} i\frac{\partial }{\partial t}\varphi_{s}\left(k_{z},t\right) & = & \left[\frac{\hbar k_{z}^{2}}{2m}-\frac{s\gamma \;B_{0}}{2}-\frac{is\gamma \;B_{1}}{2}\frac{\partial }{\partial k_{z}}\right]\varphi_{s}\left(k_{z},t\right)\;, \end{array}$$

where s=±1 denotes the eigenvalues of $\sigma^{z}$ and we omitted the constant term proportional to $k_{x}^{2}$. We eliminate the term proportional to $B_{0}$ by a transformation to a rotating frame. Substituting $\varphi_{s}\left(k_{z},t\right)=e^{ist\gamma \;B_{0}/2}\phi_{s}\left(k_{z},t\right)$, Equation (A14) becomes

(A15) $$\begin{array}{ccc} i\frac{\partial }{\partial t}\phi_{s}\left(k_{z},t\right) & = & \left[\frac{\hbar k_{z}^{2}}{2m}-\frac{is\gamma \;B_{1}}{2}\frac{\partial }{\partial k_{z}}\right]\phi_{s}\left(k_{z},t\right)\;. \end{array}$$

Changing to new variables defined by $k_{z}=k^{\prime }+gt^{\prime }$ and $t=t^{\prime }$, we have $\frac{\partial }{\partial k^{\prime }}=\frac{\partial }{\partial k_{z}}$, $\frac{\partial }{\partial t^{\prime }}=\frac{\partial }{\partial t}+g\frac{\partial }{\partial k_{z}}$, and Equation (A15) changes to

(A16) $$i\left[\frac{\partial }{\partial t^{\prime }}+\left(\frac{s\gamma \;B_{1}}{2}-g\right)\frac{\partial }{\partial k^{\prime }}\right]\phi_{s}\left(k^{\prime },t^{\prime }\right)=\frac{\hbar {\left(k^{\prime }+gt^{\prime }\right)}^{2}}{2m}\phi_{s}\left(k^{\prime },t\right)\;,$$

Choosing $g=s\gamma \;B_{1}/2$, Equation (A16) simplifies to

(A17) $$\begin{array}{ccc} i\frac{\partial }{\partial t^{\prime }}\phi_{s}\left(k^{\prime },t^{\prime }\right) & = & \frac{\hbar {\left(k^{\prime }+gt^{\prime }\right)}^{2}}{2m}\phi_{s}\left(k^{\prime },t^{\prime }\right)\;, \end{array}$$

the solution of which reads

(A18) $$\begin{array}{ccc} \phi_{s}\left(k^{\prime },t^{\prime }\right) & = & e^{-i\hbar \;t^{\prime }\left[k^{\prime 2}+gt^{\prime }+g^{2}t^{\prime 2}/3\right]/2m}\phi_{s}\left(k^{\prime },0\right)\\ & = & e^{-i\hbar g^{2}t^{\prime 3}/24m}e^{-i\hbar t^{\prime }{\left(k^{\prime }+gt^{\prime }/2\right)}^{2}/2m}\phi_{s}\left(k^{\prime },0\right)\;, \end{array}$$

or, in terms of the original coordinates,

(A19) $$\varphi_{s}\left(k_{z},t\right)=e^{ist\gamma \;B_{0}/2}e^{-i\hbar g^{2}t^{3}/24m}e^{-i\hbar t{\left(k_{z}-gt/2\right)}^{2}/2m}\varphi_{s}\left(k_{z}-gt,0\right)\;.$$

It then follows that

(A20) $$\begin{array}{ccc} |\varphi_{s}\left(k_{z}+s\gamma \;B_{1}\;t/2,t\right){|}^{2} & = & |\varphi_{s}\left(k_{z},0\right){|}^{2}\;,\;s=\pm 1\;, \end{array}$$

for any choice of the initial state $\varphi_{s}\left(k_{z},0\right)$. Equation (A20) tells us that as a function of time, the probability density $|\varphi_{s}\left(k_{z},t\right){|}^{2}$ is the same as the initial probability density, translated in momentum space by $-s\gamma \;B_{1}\;t/2$.

Assuming that $\langle \varphi_{s}\left(k_{z},0\right)|\varphi_{s}\left(k_{z},0\right)\rangle =1$, it follows from Equation (A19) that

(A21) $$\begin{array}{ccc} {\langle k_{z}\left(t\right)\rangle }_{s} & = & \langle \varphi_{s}\left(k_{z},t\right)|k_{z}|\varphi_{s}\left(k_{z},t\right)\rangle \\ & = & \langle \varphi_{s}\left(k_{z},t\right)|\left(k_{z}-gt\right)|\varphi_{s}\left(k,t\right)\rangle +gt\langle \varphi_{s}\left(k,t\right)|\varphi_{s}\left(k,t\right)\rangle \\ & = & \langle \varphi_{s}\left(k_{z},0\right)|k_{z}|\varphi_{s}\left(k_{z},0\right)\rangle +gt={\langle k_{z}\left(0\right)\rangle }_{s}+gt\\ & = & {\langle k_{z}\left(0\right)\rangle }_{s}+\frac{s\gamma \;B_{1}\;t}{2}\;. \end{array}$$

Therefore, in the textbook case, the presence of a gradient in the magnetic field causes the average momentum to linearly decrease (s=+1) or increase (s=−1) if γ<0 (which is the case for neutrons or imaginary silver particles). Integrating Equation (A21) with respect to time yields ${\langle z\left(t\right)\rangle }_{s}={\langle q_{z}\left(t\right)\rangle }_{s}={\langle z\left(0\right)\rangle }_{s}+{\langle k_{z}\left(0\right)\rangle }_{s}\;t+s\gamma \;B_{1}\;t^{2}/4$ showing that the average position traces out a parabolic trajectory [7].

Writing Equation (A21) in terms of the quantum-theoretical velocity operator defined by v=ℏk/m, we have ${\langle v_{z}\left(t\right)\rangle }_{\pm 1}={\langle v_{z}\left(0\right)\rangle }_{\pm 1}\pm \hbar \gamma \;B_{1}\;t/2m$. The classical mechanical expression $\pm \hbar \gamma B_{1}St/m$ for the change of the velocity due to the magnetic field gradient matches the quantum-theoretical result if S=1/2, as mentioned earlier.

### Appendix D.3. Dimensionless Form

We define $\hbar k_{x}/m=v_{0}v_{x}$, $\hbar k_{z}/m=v_{0}v_{z}$, and $t=t_{0}\tau$ where $v_{0}$ and $t_{0}$ set the scale of the velocity and time, respectively. In terms of these variables Equation (A12) reads

(A22) $$\begin{array}{c} i\frac{\partial }{\partial \tau }|\Phi \left(\tau \right)\rangle =\left[\frac{mv_{0}^{2}t_{0}}{2\hbar }\left(v_{x}^{2}+v_{z}^{2}\right)-\frac{\gamma \;B_{0}t_{0}}{2}\sigma^{z}-\frac{i\hbar \gamma \;B_{1}t_{0}}{2mv_{0}}\left(\sigma^{z}\frac{\partial }{\partial v_{z}}-\sigma^{x}\frac{\partial }{\partial v_{x}}\right)\right]|\Phi \left(\tau \right)\rangle \;. \end{array}$$

We simplify the notation somewhat by introducing the (dimensionless) parameters

(A23) $$\begin{array}{c} a=\frac{mt_{0}v_{0}^{2}}{2\hbar }\;,\;b=\frac{\hbar \gamma \;B_{1}t_{0}}{2mv_{0}}\;,\;c=\frac{\gamma \;B_{0}t_{0}}{2}\;, \end{array}$$

and, at the risk of creating confusion, make the replacements τ→t, $v_{x}\to x$, $v_{z}\to z$, $i\frac{\partial }{\partial v_{x}}\to p_{x}$ and $i\frac{\partial }{\partial v_{z}}\to p_{z}$. Then, Equation (A22) becomes

(A24) $$i\frac{\partial }{\partial t}|\Phi \left(t\right)\rangle =\left[a\left(x^{2}+z^{2}\right)-c\sigma^{z}-b\sigma^{z}p_{z}+b\sigma^{x}p_{x}\right]|\Phi \left(t\right)\rangle \;.$$

We emphasize that from now on, whenever we discuss the quantum-theoretical model, *x* and *z* denote the dimensionless velocity in the *x*- and *z*-direction, respectively.

In the case of neutrons, we use the parameters given in Equation (10) and the corresponding values of $t_{0}=t^{★}$ and $v_{0}=v^{★}$ to find

(A25) $$\begin{array}{ccc} a & = & 196,540\;,\;b=-1\;,\;c=-185,339B_{0}\;T^{-1}\;, \end{array}$$

whereas for c (using Equation (11)), we find

(A26) $$\begin{array}{ccc} a & = & 2.53618\;,\;b=-1\;,\;c=-8053.19B_{0}\;T^{-1}\;. \end{array}$$

Comparing Equations (A25) and (A26), we may expect that numerically solving the TDPE Equation (A24) for the case of neutrons is much more difficult than for the case of imaginary silver particles, simply because in the former *a* and *b* differ by more than five orders of magnitude.

### Appendix D.4. Initial State

The initial two-component spinor in the momentum representation is given by

(A27) $$\begin{array}{c} \langle x,z|\Phi \left(t=0\right)\rangle =\left(\begin{array}{c} \cos\left(\theta /2\right)e^{-i\alpha /2}\\ \sin\left(\theta /2\right)e^{+i\alpha /2} \end{array}\right)G\left(x,z\right)\;, \end{array}$$

where θ controls the ratio and α controls the phases of the spin-up and spin-down components, respectively. The function G(x,z) is taken to be

(A28) $$\begin{array}{ccc} G(x,z) & = & \frac{1}{2\pi \sigma^{2}}\exp\left(-\frac{x^{2}+z^{2}}{2\sigma^{2}}\right)\;, \end{array}$$

the standard Gaussian distribution with variance σ and centered around (0,0).

In practice, we either use uniform random numbers to determine cos(θ/2) and α or we set α=π/4 and choose θ from the set {π/4,−π/4,0,π/2}, corresponding to the spin states $(|↑\rangle +\left|↓\rangle \right)/\sqrt{2}$, $(|↑\rangle -\left|↓\rangle \right)/\sqrt{2}$, |↑〉, and |↓〉, respectively.

### Appendix D.5. Simulation Method

In practice, we solve Equation (A24) by means of the Chebyshev polynomial algorithm [46,47]. Disregarding the discretization of the $p_{x}$ and $p_{z}$, this algorithm has the virtue that it yields numerical results with close to machine precision, for any time *t* [46,47]. Technical details about this TDPE solver can be found in Appendix D.6.

For the reasons explained in Appendix D.6, simulating the neutron experiment [10] is prohibitively costly. A simple way out of this conundrum is to “make the SG magnet shorter” by a factor of ten. Then $t_{0}=t^{★}/10$, $v_{0}=v^{★}/10$ and, according to Equation (A23), *a* is reduced by a factor of thousand and *b* is left unchanged.

A simulation with $t_{0}=t^{*}/10$ and $v_{0}=v^{*}/10$, using a grid of $2^{15}\times 2^{15}$ points requires about 200 GiB of memory and finishes in about 8 hours, using a compute node with two AMD EPYC 7742, 2×64 cores, 2.25 GHz processors. Although it would be technically straightforward to use more nodes by extending the code to use the MPI communication protocol, we believe that this extension would not bring much additional insight. The main point here is that we can perform simulations in which the neutrons travel a macroscopic distance and the dimensions of the apparatus are also macroscopic.

It may be of interest to mention that in practice, a product-formula approach [22,48] using the decomposition in terms of the exact propagators in Equation (A10) for the *x* and *z* components fails, simply because of the large disparity between the coefficients *a* and *b* (in the case of neutrons).

### Appendix D.6. TDPE Solver: Technical Aspects

We discretize the *x* and *z* variables by using a square regular grid. The mesh size δ should be small enough to support (i) an accurate representation of terms with the first derivatives and (ii) accurately resolve the dependence of the “potential” $a(x^{2}+z^{2})$ on *x* and *z*. As |b|=1, condition (i) is automatically satisfied if (ii) is satisfied. For the neutron case, the fact that *a* is more than five orders of magnitude larger than |b| makes it difficult to satisfy condition (ii).

Ideally, for the numerical solution of Equation (A12), we would like to take $t_{0}=t^{*}$ and $v_{0}=v^{*}$ as the scales of time and velocity, respectively. We do so in the case of imaginary silver particles. Unfortunately, for the case of neutrons an accurate numerical solution of Equation (A12) for $t_{0}=t^{*}$ and $v_{0}=v^{*}$ requires a currently prohibitive amount of memory and CPU time.

To appreciate the difficulty of satisfying condition (ii) it is sufficient to focus on the *x*-dependence and consider the case $B_{0}=0$. We assume δ≪1 in the following. Initially, the wave packet is concentrated around −mδ≤x≤mδ where *m* is a small (compared to the grid size) integer. The potential of two neighboring grid points in the vicinity of x=0 differs by the amount $a{\left(x\pm \delta \right)}^{2}-ax^{2}\approx \pm a\delta^{2}\left(2m\pm 1\right)$. On the other hand, with our choice of dimensionless units, the relevant range of the dimensionless velocities *x* and *z* is approximately [−1,1]. If, in the course of time, (part of) the wave packet is concentrated around say x=1, the potential of two neighboring grid points differs by the amount $a{\left(x\pm \delta \right)}^{2}-ax^{2}\approx \pm 2a\delta$. In order to represent the smoothly changing potential $ax^{2}$ on a grid, we must require 2aδ≪1. Otherwise, when the wave moves towards x=±1, it will encounter a potential that changes in big steps and its dynamics will no longer resemble that of a wave propagating in a continuum. As explained below, the number of grid points in one direction that we use is of the order $2^{15}$, yielding a grid size of $\delta =4/2^{15}=2^{-13}\approx 0.00012$. For neutrons a=196,540, 2aδ≈48, which is much too large. In contrast, for imaginary silver particles a=2.53618, 2aδ≈0.0006, which is definitely small enough; therefore, in the case of neutrons, we are forced to reduce the size of the simulation problem.

### Appendix D.7. Quantum Dynamics: Imaginary Silver Particles

Unlike in the case of neutrons in which memory requirements limited the TDPE integration time to $t^{★}/10$, the parameters for imaginary silver particles are such that there are no such limitations. Thus, in this case $v_{0}=v^{*}$. The value of $B_{0}$ at which the two spots changes into a circular shape is hard to predict without actually performing the simulation.
